# Survival and Intra-Nuclear Trafficking of *Burkholderia pseudomallei*: Strategies of Evasion from Immune Surveillance?

**DOI:** 10.1371/journal.pntd.0005241

**Published:** 2017-01-03

**Authors:** Jamuna Vadivelu, Kumutha Malar Vellasamy, Jaikumar Thimma, Vanitha Mariappan, Wen-Tyng Kang, Leang-Chung Choh, Esaki M. Shankar, Kum Thong Wong

**Affiliations:** 1 Department of Medical Microbiology, Faculty of Medicine, University of Malaya Kuala Lumpur, Malaysia; 2 Division of Infection Biology, Department of Life Sciences, Central University of Tamil Nadu (CUTN), Thiruvarur, India; 3 Department of Pathology, Faculty of Medicine, University of Malaya, Kuala Lumpur, Malaysia; Fondation Raoul Follereau, FRANCE

## Abstract

**Background:**

During infection, successful bacterial clearance is achieved via the host immune system acting in conjunction with appropriate antibiotic therapy. However, it still remains a tip of the iceberg as to where persistent pathogens namely, *Burkholderia pseudomallei* (*B*. *pseudomallei*) reside/hide to escape from host immune sensors and antimicrobial pressure.

**Methods:**

We used transmission electron microscopy (TEM) to investigate post-mortem tissue sections of patients with clinical melioidosis to identify the localisation of a recently identified gut microbiome, *B*. *pseudomallei* within host cells. The intranuclear presence of *B*. *pseudomallei* was confirmed using transmission electron microscopy (TEM) of experimentally infected guinea pig spleen tissues and Live Z-stack, and ImageJ analysis of fluorescence microscopy analysis of *in vitro* infection of A549 human lung epithelial cells.

**Results:**

TEM investigations revealed intranuclear localization of *B*. *pseudomallei* in cells of infected human lung and guinea pig spleen tissues. We also found that *B*. *pseudomallei* induced actin polymerization following infection of A549 human lung epithelial cells. Infected A549 lung epithelial cells using 3D-Laser scanning confocal microscopy (LSCM) and immunofluorescence microscopy confirmed the intranuclear localization of *B*. *pseudomallei*.

**Conclusion:**

*B*. *pseudomallei* was found within the nuclear compartment of host cells. The nucleus may play a role as an occult or transient niche for persistence of intracellular pathogens, potentially leading to recurrrent episodes or recrudescence of infection.

## Author Summary

*Burkholderia pseudomallei* (*B*. *pseudomallei*), the causative agent of melioidosis, is endemic across parts of South East Asia and Northern Australia. Of the key features of *B*. *pseudomallei*, is its ability to remain latent in the host causing recrudescent disease years after initial infection. However, it still remains unclear as to where *B*. *pseudomallei* resides to escape from host immune sensors and antimicrobial pressure. Here, we have found that *B*. *pseudomallei* was able to enter into the nuclear compartment of host cells. The nucleus may play a role as a temporary abode for persistence, leading to recurrrent episodes of infection.

## Introduction

Mammals including human harbour a complex gastrointestinal microbiota which forms an extraordinary symbiosis with each other. The microbiota may be influenced by diet, developing immune system, chemical exposures and, potentially, the founder effects of initial colonizers. The early colonizers are thought to have long-term effects on the establishment of the microbiota. In a recent study by our group, we isolated melioidosis-causing Gram-negative bacillus *Burkholderia pseudomallei* from a group of *Helicobacter pylori*-positive gastric patients [1]. Melioidosis is a fatal tropical infectious disease with a myriad of clinical symptoms which impose difficulties for clinicians in differential diagnosis for other common infectious diseases [2]. The stomach, where the *B*. *pseudomallei* strains were isolated from, is not reported as a natural colonization niche and isolation site for *B*. *pseudomallei* [1]. More strikingly, the patients did not present with the symptoms of melioidosis.

*B*. *pseudomallei* is able to cause recrudescing or latent infections in the host. The bacterium is a category B biothreat agent as classified by the Centers for Disease Control, and has been demonstrated to have an intracellular lifestyle causing invasive melioidosis in man and animals. The environmental niche for this bacterium is the soils and contaminated water of Southeast Asia and northern Australia, and infection is acquired via inhalation, ingestion and inoculation through the abraded skin and mucosa [3]. The clinical presentation ranges from fever and acute fulminant community-acquired pneumonia to fatal abscess and septicaemia. In spite of intensive antimicrobial therapy and high antibody titers, *B*. *pseudomallei* can remain latent and cause recrudescence or relapses following years of primary infection in the host [3, 4]. Despite the remarkable progress in medical science over the last few decades largely owing to advances in cutting-edge research technologies, persistent bacterial infections including recrudescing or latent infections continues to remain an unresolved challenge and unsolved puzzle in the field of microbiology.

A common characteristic of many bacterial infections in human is the ability of the host immune system to effectively eliminate the invading bacteria and resolve the infection, which can normally be achieved via the extracellular defense mechanisms namely, antimicrobial peptides, complement and antibodies; and/or the intracellular immune mechanisms including reactive oxygen and nitrogen intermediates, and lysosomes [5]. The use of appropriate antimicrobial therapy may also aid eradication of infection. However, in the case of persistent and recrudescing infections, the invading bacteria appear to withstand host pressure despite the onset of inflammation, specific antimicrobial immune mechanisms, and appropriate antimicrobial therapy [6]. Thus, contrary to acute bacterial infections, eradication of persistent infections remains a challenge and continues to account for significant rates of morbidity and mortality. Experimental evidence suggests that viral and bacterial pathogens reportedly invade the host intracellular compartment via microtubule- or actin polymerization-dependent mechanisms [7, 8]. Following intracellular entry, motility offers bacteria an opportunity to invade the nucleus via transit through pore complexes present on the nuclear envelope [8]. A broad array of bacterial and viral target proteins are known to co-localize in the perinuclear [9, 10], and nuclear regions [11] of host cells. Localization of viruses in the intranuclear compartment of host cells have also been reported [12]. However, only a few intracellular bacteria such as *Holospora* spp., [13] *Rickettsia rickettsii*, and *Rickettsia bellii* [14] have been documented to have localized the intranuclear compartment till date.

Several cytological studies have established the ability of *B*. *pseudomallei* to escape into the cytosol from endocytic vacuoles, where it polymerizes actin and subsequently invades adjacent cells [15–17]. This suggests that *B*. *pseudomallei* adapt to a quiescent state in an intracellular location to escape from exuberant host immune surveillance. Therefore, it is important to explore the intracellular localization of persistent bacteria to be able to achieve complete clearance and control of intracellular infections. Hence, we asked if *B*. *pseudomallei* could be trafficked into the nucleus as a survival strategy.

## Results

### Transmission electron microscopy (TEM) investigations revealed intranuclear localization of *B*. *pseudomallei* in cells of infected human lung and guinea pig spleen tissues

Ingress and egress of *B*. *pseudomallei* from the cytoplasm of eukaryotic cells reportedly induces actin rearrangement [17]. Similar mechanisms have been reported to occur in *Holospora* spp., which are known to polymerize and depolymerize actin fibrils to encroach the nucleoplasm of host cells [18]. This, in a way, helps bacteria to invade the host nucleus to persist intracellularly. Hence, we hypothesized if a similar phenomenon occurs in *B*. *pseudomallei* whereby it could gain access into the nucleosol. To investigate this, we obtained tissue samples of human lungs on post-mortem following death of two patients with clinical melioidosis, and spleen of eight experimental guinea pigs infected subcutaneously with 10^6^ cfu/ml of *B*. *pseudomallei* CMS strain, from sites with abscesses following death of the animal. Subsequently, the tissue sections obtained were confirmed for presence of *B*. *pseudomallei* using TEM examination.

**Fig 1:**
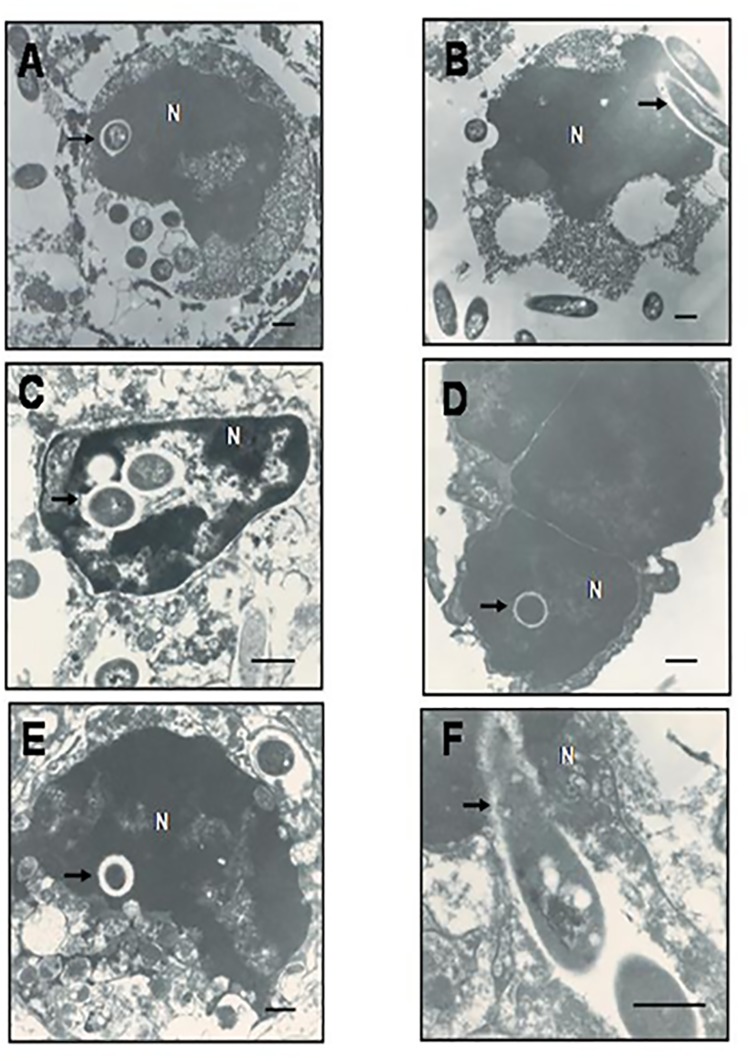
Transmission electron micrographs of human lung and guinea pig spleen infected with *B*. *pseudomallei*. (**1A & 1B**) Human lung tissue sections derived from autopsy specimens of clinical melioidosis (magnification: 10000X). (**1C – 1F**) Splenic abscess tissue sections derived following infection of guinea pigs with *B*. *pseudomallei* (Magnification: **C**, 17000X; **D** & **E**, 13000X & **F**, 28000X). The arrows indicate the intranuclear localization of *B*. *pseudomallei* (**B & F**). (**A, C, D & E**) The arrows indicate the intranuclear localization of *B. pseudomallei*. (**B & F**) The arrow indicates the entry of bacteria into the nucleus. Bars represent 500nm.

Our investigations showed that *B*. *pseudomallei* occupied a significant portion of the cytoplasm of cells of infected human lung (**Fig 1A**) and guinea pig spleen (**Fig 1C**) tissues. The bacteria were highly uniform in shape and size with structural characteristics consistent to Gram-negative bacillus that were later confirmed as *B*. *pseudomallei* using immunohistochemical methods. Strikingly, we also visualized localization of *B*. *pseudomallei* in the nucleoplasm of the cells of human lung and guinea pig spleen tissues (**Fig 1A–1E**). An electron-lucent zone (“halo zone”) surrounding the bacteria was also observed, which is in line with previous findings of an extracellular matrix (capsular) zone demonstrated using electron microscopic techniques [13], providing additional confirmation to existence of *B*. *pseudomallei* in the nucleus. We also observed apparent invasion of *B*. *pseudomallei* into the nuclear region of cells extracted from human lung (**Fig 1B**) and guinea pig spleen tissues (**Fig 1F**). However, owing to the necrotic nature of tissues collected from abscess sites, we could see limited integrity in the cellular and nuclear membranes. The presence of intact halo surrounding the bacterium (capsule) clearly delineates the nucleoplasm, and demonstrates that the bacterium clearly occurred within the nucleus. Hence, our findings convincingly showed the intranuclear existence of B. *pseudomallei* in cells of human lung and guinea pig spleen tissues.

### *B*. *pseudomallei* induced actin polymerization following infection of A549 human lung epithelial cells

**Fig 2:**
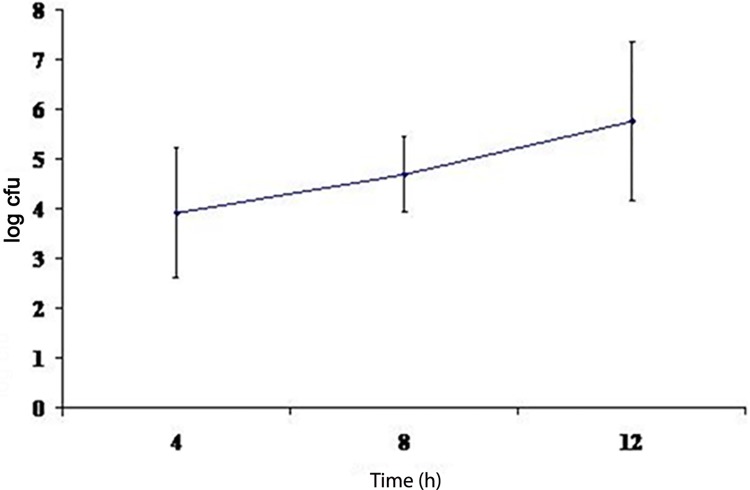
Intracellular survival of *B*. *pseudomallei* K96243 in A549 human lung epithelial cells determined following 4, 8 and 12 hours of infection.

**Fig 3:**
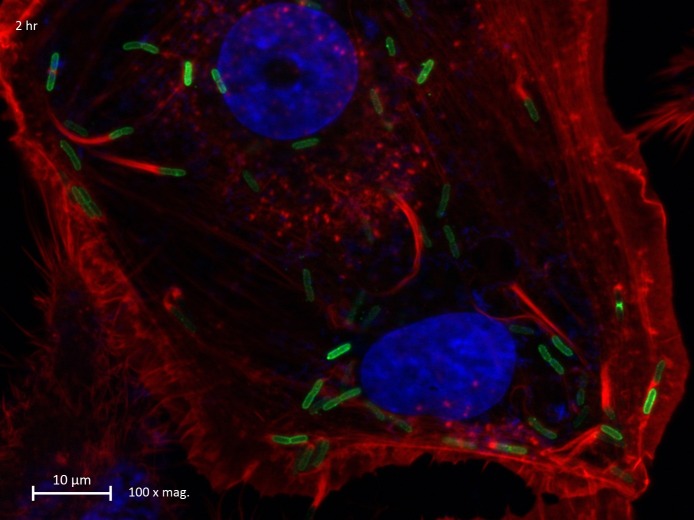
Fluorescence photomicrograph of membrane protrusion of actin tail in A549 human lung epithelial cell following *B*. *pseudomallei* infection. Figure represents the presence of membrane protrusion with typical actin tail (arrow) that occurred 4 hours following infection with *B. pseudomallei* K96243. Actin fibres were stained red, nuclei were stained blue and the bacteria were stained green.

Next, we sought to investigate the mechanism that the *B*. *pseudomallei* utilizes to invade the intracellular compartment and whether it employs actin polymerization to enter the cell. Direct bacterial spread is accomplished via recruitment of host cell cytoskeleton. Certain bacteria, for instance, *Shigella* spp. and *Listeria monocytogenes* induce actin polymerization at one of their ends forming a cytoskeletal scaffold, and move forward as actin is eventually polymerized and depolymerized rapidly at the tip of the bacterium [19]. Following arrival at the cell membrane, the bacteria display finger-like projections that extend into adjacent cells, and subsequent synthesis of membrane-damaging enzymes by the bacteria helps its successful invasion into the cytoplasm of the new host cell. We initially investigated the ability of *B*. *pseudomallei* strain K96243 to invade and survive intracellularly in A549 human lung epithelial cells. A mean of three independent invasion assays were used to calculate the percentage of invasion of *B*. *pseudomallei* K96243 in the cells that showed (3∙95± 2∙40) X 10^−1^% invasion. An intriguing observation was that *B*. *pseudomallei* K96243 was able to replicate intracellularly in A549 cells with the number of intracellular bacteria steadily increasing over time (**Fig 2**). This finding was strongly supported by the observation that *B*. *pseudomallei* induced actin polymerization in the infected A549 lung epithelial cells. When the infected cells were stained with rhodamine-conjugated phalloidin for actin fibers, actin rearrangement was visualized as a comet-like trail behind the bacterium, with actin polymerizing at the “head” and depolymerizing along the “tail” (**Fig 3**). Hence, we found that *B*. *pseudomallei* induces actin polymerization in infected A549 human lung epithelial cells.

### Infected A549 lung epithelial cells using 3D-Laser scanning confocal microscopy (LSCM) and immunofluorescence microscopy confirmed the intranuclear localization of *B*. *pseudomallei*

The presence of *B*. *pseudomallei* within the nucleoplasm needs to be further confirmed as a real event and not due to artefacts or effect of sectioning and processing of the samples. Therefore, we set forth to further confirm the encroachment of nucleoplasm of living intact cells by *B*. *pseudomallei*. We cultured A549 cells infected with *B*. *pseudomallei* K96243 and examined for the potential intranuclear localization of *B*. *pseudomallei* using LSCM analysis. The cells were delineated by staining the nuclear compartment with 4',6-diamidino-2-phenylindole (DAPI) to enable identification of the intracellular plane during optical sectioning. Our investigations revealed the presence of intracellular bacteria within the nuclear compartment of the infected A549 cells. Although copious numbers of bacteria were found to occur in the cytoplasm, a few were also seen within in the nucleus.

**Fig 4:**
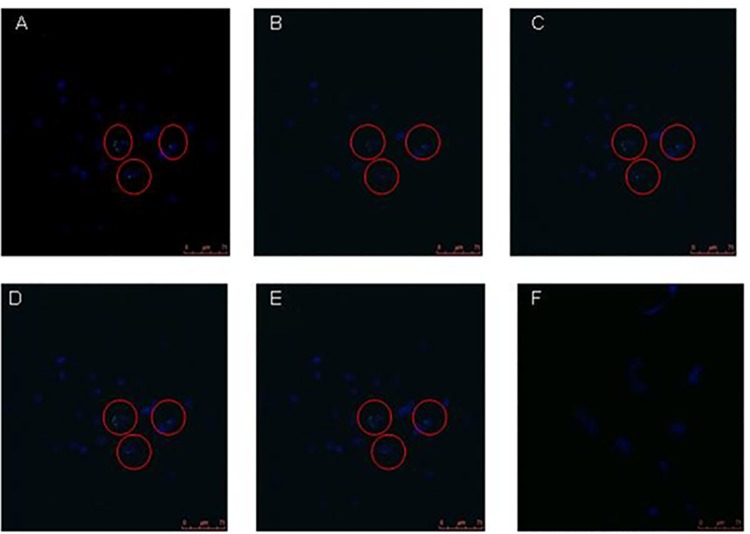
Laser scanning confocal microscopy (LSCM) of *B*. *pseudomallei*-infected A549 human lung epithelial cells. (**4A – 4E**) Z-stacks of *B*. *pseudomallei*-infected A549 human lung epithelial cells (images captured under 40X objective). The 4',6-diamidino-2-phenylindole- (DAPI) stained A549 cell nucleus appears blue, whereas the greenish *B*. *pseudomallei* were visualized using primary *B*. *pseudomallei*-specific and Alexa fluor 488-conjugated secondary antibodies. (**4F**) Negative control of uninfected A549 human lung epithelial cells. DAPI, 4',6-diamidino-2-phenylindole; LSCM, laser scanning confocal microscopy.

**Fig 5:**
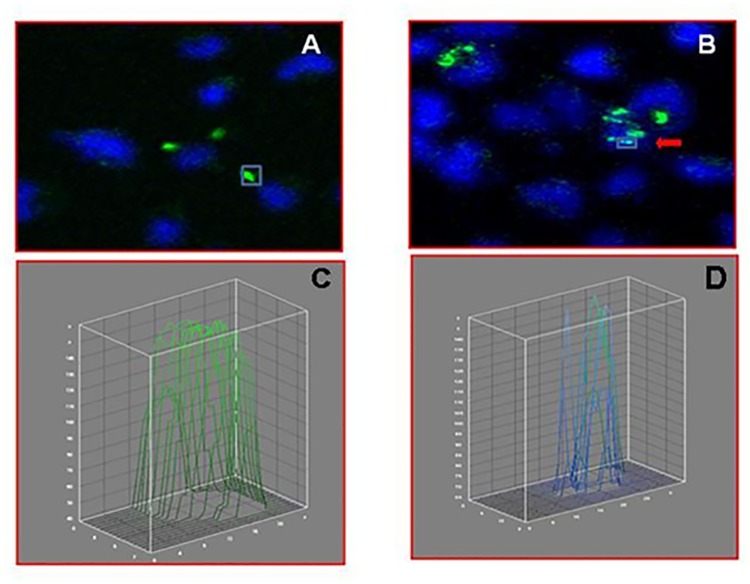
Interactive 3D surface plot for the confirmation of *B*. *pseudomallei*-associated immunofluorescence emanating from within the volume of nucleus. Fluorescence distribution of Alexa fluor 488-stained *B*. *pseudomallei* is depicted as green line whereas the fluorescence distribution of 4',6-diamidino-2-phenylindole (DAPI) is depicted as blue line. Bacteria present in different sections were selected, and fluorescence intensities were depicted graphically using interactive 3D surface plot images. The fluorescence intensities were interpreted as height for the plot in this software. (**5A & 5C**) *B*. *pseudomallei* shown localized within the cytoplasmic compartment and its corresponding 3D plot. (**5B & 5D**) *B*. *pseudomallei* shown ensconcing within the nuclear compartment and its respective 3D plot images. The images were analyzed using ImageJ software (http://rsb.info.nih.gov/ij/) to confirm the ensconcing *B*. *pseudomallei* within the nucleus. DAPI, 4',6-diamidino-2-phenylindole.

To further strengthen our finding of nuclear trafficking of *B*. *pseudomallei*, we evaluated five images that were captured using a Z-stack analysis (**Fig 4A–4E**). Our results evidently showed that *B*. *pseudomallei* were clearly seen ensconced within the infected host cell nucleus. Appropriate negative controls of uninfected A549 cells were included (**Fig 4F**) to show the specificity of green fluorescence where only the bacteria could be stained using green fluorescence. ImageJ analysis generated for specific regions also confirmed the localization of *B*. *pseudomallei* within the nucleus. The fluorescence intensity within the region of bacteria localized outside the nucleus yielded only green lines (**Fig 5A & 5C**) whereas the region occupied by the intranuclear bacteria divulged green together with blue lines (**Fig 5B & 5D**). The green line was entirely contained within the area occupied by the DAPI-stained host nucleus. Therefore, our findings strongly showed that *B*. *pseudomallei* is found within the host cell nucleus.

**Fig 6:**
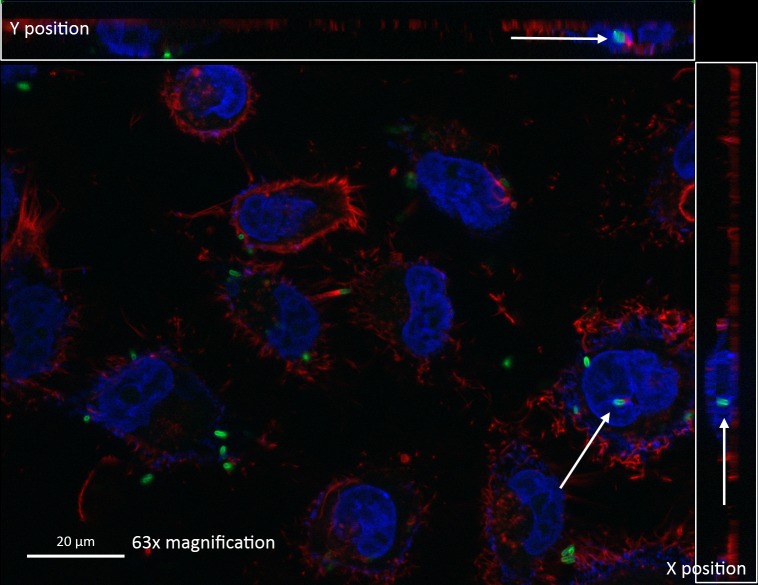
Vertical section of A549 human lung epithelial cells harboring *B*. *pseudomallei* within the nucleus. Arrows indicate the presence of *B. pseudomallei* within the nuclear compartment of infected cells. Actin fibres were stained red, nuclei were stained blue and the bacteria were stained green.

To further strengthen our observations, vertical sectioning of fluorescence microscopy images were examined, which clearly established the presence of the fluorescein isothiocyanate (FITC)-stained bacteria (green) within the DAPI-stained nucleus (blue) of A549 lung epithelial cells (**Fig 6**). Additionally, the Z-stack video images obtained using immunofluorescence microscopy provides additional proof to the trafficking of *B*. *pseudomallei* into the nuclear compartment (**Supplementary S1 Video**).

## Discussion

Persistent infections represent a major global challenge for humankind, claiming millions of lives every year as well as demanding huge medical and social resources. During the development of persistent infections, the host and the pathogen play a game of ‘hide and seek’, whereby the host tries to locate and eliminate the pathogen while the pathogen strives to survive in the host. The ultimate goal of every pathogen is not to kill the host but to survive and replicate. In order for it to overcome the formidable host defense armory, the pathogen is endowed with virulence traits, such as attachment, invasion and transmission. *B*. *pseudomallei* and other intracellular pathogens, with the ability to cause persistent infection, reportedly exploit a vast range of niches within their hosts including the phagosome and cytosol [20]. *B*. *pseudomallei* persist for decades causing overwhelmingly severe relapses and recrudescence in over one-third of survivors of severe melioidosis [21]. Notwithstanding the persistent presence of high titers of antibodies for years, these survivors appear to succumb from recrudescent melioidosis, largely owing to the intracellular adaptation of *B*. *pseudomallei*, which protects it from exuberant host immune surveillance attributes namely, complement proteins, phagocytes, effector T cells, and circulating antibodies [20]. In addition, sustenance of high antibody levels suggest either persistent exposure to bacteria or bacterial antigens sequestered from an intracellular niche or occult site, thus lending ample support to the concept that the organism is indeed present in the host [22]. Experiences with human studies suggest that antimicrobial therapies are seemingly ineffective against *B*. *pseudomallei* infections, and that this sabotage is partly attributed to poor permeability of antimicrobials into the intracellular compartment [15, 20]. Therefore, we asked the question “then where else could *B*. *pseudomallei* ensconce within the host cell?”

*B*. *pseudomallei* has the ability to infect both phagocytic and non-phagocytic cells during their life cycle, [13] and cellular pathogenesis of *B*. *pseudomallei* infection involves attachment, entry, and internalization within membrane-bound vacuoles, which progressively acidifies and develops into a mature degradative phagolysosome [23]. *B*. *pseudomallei* survive the phagolysosome either by preventing vacuole–lysosome fusion or by modifying the acidic environment within the phagolysosome. The bacterium has also evolved to escape from the vacuole and survive within the cytosol [17]. In the cytosol, rich source of nutrients and protection from extracellular immune factors are rendered for the survival of *B*. *pseudomallei*. In the present study, examination of human tissue autopsy sections using TEM convincingly established the presence of *B*. *pseudomallei* within membrane-bound vesicles of both cytoplasm and nucleus.

To further prove nuclear trafficking of *B*. *pseudomallei* was not accidental due to pre-existent necrotic cells or oxidative injury caused by the intracellular bacteria that could have led to altered membrane permeability [24], we examined splenic tissue biopsy sections derived from experimentally-infected guinea pigs using TEM. The ensconcing of *B*. *pseudomallei* within membrane-bound vesicles of both cytoplasm and nucleus of tissue biopsy of guinea pigs was clearly evident. This further fortified the hypothesis that the nucleus may act as a protective niche or a temporary hiding to aid bacterial persistence. However, presence of *B*. *pseudomallei* within the nucleoplasm needed to be confirmed as indeed a true event, and not due to the effect of sectioning artefacts during the processing of samples for TEM. Therefore, we examined experimentally-infected A549 human lung epithelial cells using LSCM and 3D imaging systems to further confirm the presence of *B*. *pseudomallei* in the host nucleus. Initial invasion and intracellular survival assays were performed to determine the ability of *B*. *pseudomallei* to invade and survive intracellularly in A549 cells followed by LSCM observation of these cells. Analysis of LSCM images using Z-stack and the interactive 3D surface plot ImageJ analysis strongly underpinned the association of *B*. *pseudomallei* within the nuclear region as seen clearly from the live imaging investigations.

**Fig 7:**
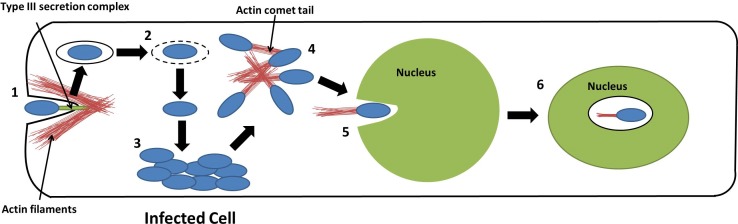
Model for entry and localization of *B*. *pseudomallei* in the nucleus of host cell. [1] Entry of *B*. *pseudomallei* into the epithelial cell aided by the type III secretion system (TTSS), which induces the formation of membrane ruffles necessary for uptake of the bacteria, [2] *B*. *pseudomallei* escapes from the endosome into the cytoplasm of the cell, [3] *B*. *pseudomallei* replicate intracellularly in the cytoplasm of the host, [4] *B*. *pseudomallei* polymerizes host cell actin to form actin comet tails in order for it to mobilize within host cell cytoplasm, [5] *B*. *pseudomallei* enters the nucleus of host cell via actin comet tails, and [6] Localization of *B*. *pseudomallei* in the nucleus of the host, potentially in a dormant state.

Here, we have demonstrated the localization of *B*. *pseudomallei* in the nucleus of infected cell. The conundrum as to whether the bacterium invades the nucleus to a) obtain a protective environment from degeneration of host cells, b) evade from intrinsic restriction factors and c) render ‘pass-over’ to newer cells in order for its prolonged survival within the host, remains a gray area of investigation. In support of this, it has recently been proposed that bacteria could merely invade nucleus to influence host cellular physiology [25]. Such a hypothesis is not far from reality as bacterial entry into the host nucleus has already been reported for *Holospora* spp., an intracellular bacterial parasite that forms a symbiotic-like relationship with the host *Paramecium* spp., by invading the macro- and micronuclear compartments of the free-living ciliate [13]. Endonuclear presence of other intracellular bacteria such as *Mycoplasma genitalium* [11], *M*. *fermentans* and *Rickettsia slovaca* [26], *R*. *rickettsii*, *and R*. *bellii* [14] has also been observed. Others have proposed that *Holospora* spp. invades the nucleus via fusion of phagosomes with nuclear envelope [27]. Recent body of data strongly support the utilization of actin-based motility by *Holospora* spp. to traverse into the nucleus from cytosol [18]. It has been well established that *B*. *pseudomallei* induce actin rearrangement to enter and escape from eukaryotic cells, and also to shuttle within the cytoplasm. Hence, we speculate that similar to *Holospora* spp., *B*. *pseudomallei* could recruit actin polymerization and depolymerization to invade the nucleus, and to ease persistence for prolonged periods of time in the host. Nevertheless, nuclear localization could also be unintentional as the nucleus might not be the direct target of *B*. *pseudomallei*. Once inside the nucleus, *B*. *pseudomallei* may remain dormant in the highly viscous nucleoplasm due to presence of capsular polysaccharides or ‘steal’ the many substances dissolved in the nucleoplasm, such as nucleotides and enzymes for replication and survival. The postulated model for entry and localization of *B*. *pseudomallei* in the nucleus of the host cell is schematically summarized in **Fig 7**. The recent rebirth of suppressor T cells and immunosuppression strategies adopted by persistent infectious agents (*L*. *monocytogenes* [28], *M*. *tuberculosis* [29], human immunodeficiency virus (HIV)-1 and hepatitis C virus (HCV) to overcome exuberant cellular immune responses) have opened up newer prospects to better understand the mechanistic aspects of cellular immunopathogenesis of melioidosis. There is growing evidence to prove that the host cell cytosol provides only a limited number of nutrients and presents the bacteria with the formidable challenge of evading the different pathogen recognition mechanisms. It is imperative to explore if *B*. *pseudomallei* programs this attribute by berthing itself into the nuclear compartment. Hence, we anticipate that future elucidation will further deepen our understanding of the intranuclear localization of *B*. *pseudomallei* and thus, providing additional clue to the intracellular persistence of bacterial pathogens.

## Materials and Methods

### Ethical approval

All experiments involving human were performed in accordance with relevant guidelines, regulations and examination by the Medical Ethics Committee (MEC) of University Malaya Medical Centre (UMMC), Kuala Lumpur, Malaysia, and was conducted as per the guidelines of the International Conference on Harmonization Guidelines and Declaration of Helsinki. Tissue samples of human lungs were obtained on post-mortem following death of two melioidosis patients at the UMMC. Written informed consent was obtained from the next of kin for diagnosis purposes. The institutional ethics committee of UMMC approved use of the histological materials for this retrospective investigation (Ref. No.: 896.47). Animals experiments and protocols were performed following approval of animal ethics committee of Institutional Animal Care and Use Committee (IACUC), UM following their guidelines [Ref. No.: PAT/05/11/2007/WKT (R)] and MP/08/02/2005/JSU (M)], according to the policies of the Malaysian Animals Act 1953 (Act 647) guidelines (http://caexpo.gxciq.gov.cn/html/2011-01/346.htm). Eight guinea pigs and six BALB/c mice purchased from the Laboratory Animal Centre, University of Malaya were used in the study.

### Bacteria

*B*. *pseudomallei* strain K96243 and CMS was used in the current investigation. The isolates used were confirmed using standard laboratory methods, a commercial API 20NE system (Biomerieux, France) and culture of the isolates on nutrient agar at 37°C for 24 hours. Subsequently, a single colony of bacterial growth was inoculated into 10 ml of Luria-Bertani (LB) broth and incubated at 37°C for 18 hours with shaking [4]. The number of colony forming units (cfu) was enumerated by plating the serial dilution of the culture on nutrient agar.

### Animals and experimental inoculation

Eight guinea pigs purchased from the Laboratory Animal Centre, University of Malaya were experimentally infected subcutaneously with 10^6^cfu/ml *B*. *pseudomallei* CMS, and spleen tissues were obtained from sites with abscesses following death of the animal.

### Transmission electron microscopy

Tissue samples of human lungs and spleen tissues of guinea pigs experimentally infected with *B*. *pseudomallei* CMS, were subjected to TEM investigations. Briefly, tissue sections of 5 μm were placed on poly-L-lysine coated glass slides, dewaxed, rehydrated and washed under running tap water for five to 10 minutes. Sections of all tissues obtained were confirmed for the presence of *B*. *pseudomallei* using modified peroxidise-anti-peroxidase immunohistochemical methods. Briefly, tissue sections were fixed in 4% glutaraldehyde after which post-fixation was performed using 1% osmium tetroxide (one hour), dehydrated in sequential ethanol series, embedded in epon resin and allowed to polymerize at 60°C for 18 hours. The polymerized blocks were trimmed and ultrathin sections of 90nm were made with a diamond knife in an Ultracut E Reichert-Jung ultra-microtome, collected on copper-palladium grids, contrasted with uranyl acetate and lead citrate, and illuminated under a Hitachi H7500 electron microscope (Electron Microscopy Laboratory, Faculty of Medicine, University of Malaya).

### *B*. *pseudomallei*-specific monoclonal antibodies

Monoclonal antibodies (mAbs) were raised against concentrated culture filtrate antigens of *B*. *pseudomallei* CMS. Immunization of 6–8 weeks old BALB/c mice, production of hybridomas and fusion with Sp2 myeloma cells were performed by standard procedures. Sera from the immunized mice and tissue culture supernatants from hybridized cells were screened for reactivity by ELISA as described by Chenthamarakshan *et al*. (2001) [4].

### Invasion and intracellular survival assays

The invasion assay was performed as described by Mariappan et al. (2013) [30] with slight modifications. Briefly, confluent monolayers of human lung epithelial cells, A549 (ATCC CCL-185) (5 X 10^5^) cells in a 24-wells tissue culture plate were infected with the bacterial inoculum at a multiplicity of infection (MOI) of 1:10. Subsequently, the plates were incubated for 2 hours at 37°C in 5% CO_2_ environment to allow invasion. The monolayers were washed three times using phosphate buffered saline (PBS) (pH 7.0), followed by addition of RPMI1640 medium (Flowlabs, Australia) containing a cocktail of ceftazidime (250 μg/ml) and imipenem (250 μg/ml) into wells for two hours at 37°C to kill residual extracellular bacteria. Subsequently, the cell monolayers were washed three times with PBS and lysed using 0.25% Triton X-100. Serial dilutions of the lysate were plated onto nutrient agar to determine bacterial counts, and the percentage of invasion was calculated. A similar protocol was followed in order to determine the ability of *B*. *pseudomallei* K96243 to replicate intracellularly. However, the number of bacteria released was only calculated after a further 4, 8 and 12 hours of incubation in the culture medium. At each time point, the infected monolayers were washed and lysed using 0.25% Triton X-100, and the intracellular bacteria released were enumerated by plating the serial dilution of the lysate on nutrient agar.

### LSCM of A549 cells infected with *B*. *pseudomallei*

The A549 cells were maintained in Roswell Park Memorial Institute (RPMI) 1640 medium supplemented with 10% foetal bovine serum (FBS) (Sigma, USA), 1mM penicillin-streptomycin (Flowlabs, Australia), 2mM glutamine (Flowlabs, Australia) and the cells were grown in 5% CO_2_ incubator at 37°C. Fluorescence staining of bacteria was performed as described by others with minor modifications [16]. Briefly, the A549 cells were cultured in 8-well Lab-Tek chamber slide tissue culture chambers (Nunc, USA), and incubated for 12 hours at 37°C. Subsequently, the cells were infected with *B*. *pseudomallei* K96243 at an MOI of 1:10 for two hours, after which the cells were washed with PBS (3X) and the extracellular bacteria were killed by incubating the cells for two hours using imipenem 250μg/ml plus ceftazidime 250μg/ml cocktail in RPMI 1640 medium. Subsequently, the cells were washed three times with PBS and fixed with 3.7% paraformaldehyde in PBS for 15 minutes. Later, the cells were permeabilised with 0.1% triton X-100 for five minutes and blocked with 1% bovine serum albumin (BSA) for 30 minutes to minimize non-specific binding. The permeabilised infected cells were once again incubated for an hour with a 1:200 dilution of *B*. *pseudomallei*-specific mice C122 mAb, as determined previously. The cells were then washed with 3X PBS and incubated for an hour with a secondary antibody, Alexa fluor 488 goat anti-mouse IgG (Invitrogen). Following this, the cells were washed with 3X PBS and counterstained with DAPI (Molecular Probes, Oregon, USA) for five minutes (a stain for delineating the nucleus of eukaryotic cells) or DAPI followed by rhodamine-conjugated phalloidin (Molecular Probes, Oregon, USA) to stain actin fibers. The cells were washed once again with 3X PBS, mounted with glyceryl gel, and the stained cells were observed using a LSCM with TCS SP5 imaging system and a DMI 6000B microscope (Leica, Germany). The 40X objective under oil-immersion was used for all the datasets. Five fields were examined for intracellular bacteria in each chamber, taking precautions not to count the same field twice or more. The intranuclear localisation of *B*. *pseudomallei* was confirmed by Z-stack images. A549 cells without added bacteria were used as negative control.

The images obtained were further analyzed using ImageJ software (http://rsb.info.nih.gov/ij/) to confirm the ensconcing *B*. *pseudomallei* within the nucleus. ImageJ was used to measure the fluorescence intensity pixels from select portions of the images and plotted as 3D graph (interactive 3D surface plot), which enables the intensity pixels to be viewed in XY, XZ and YZ dimensions. The fluorescence distribution of Alexa fluor 488-stained *B*. *pseudomallei* was seen as a green line whereas that of DAPI seen as blue line. The presence of *B*. *pseudomallei* within the nuclei was determined by evaluating the selected portion in interactive 3D surface plot, and was considered to be intranuclear when the distribution of green line was within the blue line.

### Immunofluorescence microscopic analysis

The immunofluorescence staining of *B*. *pseudomallei*-infected A549 cells was performed. In brief, the A549 cells were seeded on sterile 1.3 mm glass coverslips in a 12-well plate (Corning, New York) and allowed to grow to 70% confluency. Later, the cells were infected with *B*. *pseudomallei* K96243 at a MOI of 50:1 for 2 hours, and incubated in RPMI 1640 medium with added kanamycin (2000 μg/ml) for 2 hours. Subsequently, the medium was discarded and replaced with fresh RPMI 1640 (with 200 μg/ml kanamycin) medium. Six hours later, the infected cells were fixed with 4% fresh paraformaldehyde for 1 hour followed by blockade using 0.5% (w/v) BSA in PBS for 30 minutes. Subsequently, the fixed infected cells were stained with anti-*B*. *pseudomallei* lipopolysaccharide (LPS) mouse IgG (Santa Cruz, California, USA) for 1 hour followed by FITC-conjugated anti-mouse IgG polyvalent IgG staining for 1 hour for intracellular *B*. *pseudomallei*, rhodamine-conjugated phalloidin (Molecular Probes, Oregon, USA) for 15 minutes for actin cytoskeleton staining and DAPI for 5 minutes to stain the nucleus. The samples were then mounted on glass slides by using a ProLong Gold anti-fade mounting agent. Later, the slides were viewed with Axio Imager.Z2 with ApoTome.2 (Carl Zeiss, Oberkochen, Germany). The images captured were analyzed using the Zen software (Carl Zeiss, Oberkochen, Germany).

## Supporting Information

S1 Video Z-stack video images obtained using immunofluorescence microscopy clearly showing the presence of *B*. *pseudomallei* within the nuclear compartment. (MP4)
